# ReLU-FCM trained by quasi-oppositional bare bone imperialist competition algorithm for predicting employment rate

**DOI:** 10.1371/journal.pone.0272624

**Published:** 2022-08-18

**Authors:** Aihua Guo

**Affiliations:** LiRen College, Yanshan University, Qinhuangdao, Hebei, China; Beijing University of Posts and Telecommunications, CHINA

## Abstract

Fuzzy cognitive maps (FCMs) are a powerful tool for system modeling, which can be used for static and dynamic analysis. However, traditional FCMs are usually learned by gradient-based methods, and the adopted sigmoid nonlinear activation function frequently causes gradient saturation. These two shortcomings set a limit on the modeling accuracy. To overcome those problems, we propose in this paper a new FCM with two improvements. First, the rectified linear unit (ReLu) activation function is adopted to replace the sigmoid function. Second, a newly proposed quasi-oppositional bare bone imperialist competition algorithm (QBBICA) is used to learn the FCM. The improved FCM is used to predict the employment rate of graduates from Liren College, Yanshan University. Experimental results show that the improved FCM is effective in employment rate prediction.

## 1 Introduction

Improving the employment rate and employment quality of university graduates has been an important task for China’s education authority and universities. Based on the employment status of graduates, the education authority can adjust relevant policies and the university’s faculty can adjust their education and training plans to enhance the graduates’ employability. To guarantee sufficient employment of graduates, the responsible decision-makers need to make adjustments ahead of time. Adequate supporting data is necessary to ensure that the decisions are made in a scientific manner. Hence, accurately predicting the employment rate of graduates becomes an important topic of research.

Since the employment rate of graduates is affected by many factors, accurate prediction of graduates’ employment rate remains a difficult task. In the past years, many researchers have paid much attention to the prediction of employment rate of graduates. In [1], Wang adopted a neural network with an adaptive learning back-propagation framework to construct an intelligent employment rate prediction model. In [2], Guo et al. developed a framework called Multi-major Employment Status to predict graduates’ employment status when bias exists. In the framework, a long short term memory (LSTM) with dropout was adopted as the predictor. In [3], an employment rate prediction model was constructed using the back propagation (BP) neural network based on employment rate data from 2002 to 2012. Usually, the BP neural network is trained using the gradient descent algorithm, which can be easily trapped in a local optimum. To overcome this drawback, Jiang et al. [4] proposed to use an artificial fish swarm algorithm (AFSA) to train a BP neural network for predicting university graduates’ employment rate. Zhang proposed to predict university graduates’ employment rate using regression analysis, and they constructed two regression models, i.e., the polynomial regression model and the multiple linear regression model [5]. To improve the prediction accuracy, a model that combines the B-mode relational degree and general induced order weighted averaging aggregation (GIOWA) operator was proposed in [6]. A decision tree model for employment prediction was presented in [7].

Despite much research progress in the prediction of graduates’ employment rate, there are still improvements to be made in the prediction accuracy. The reason for this problem is twofold. First, the employment rate data of a single university is not large enough. That is to say, one can not obtain enough samples to construct an artificial neural networks (ANN) based prediction model. As a consequence, the learned ANN model is under or over fit and the prediction accuracy decrease. Second, factors affecting the employment rate of graduates and the causality between these factors and the employment rate of graduates are still unknown. In other words, the employment rate data contains much uncertainties. The above mentioned neural networks and gray model based prediction methods are still weaker in handling uncertainty. Therefore, it is necessary to develop an alternative model that can handle different uncertainties and perform prediction with limited data.

Fuzzy cognitive maps (FCMs), which was first introduced by Kosko [8], are a powerful tool for modeling and simulating complex dynamic systems. In nature, an FCM is a directed graph consisting of a set of nodes and edges. The nodes stand for concepts, variables or factors in a system, and they are connected with each other through edges. The weights of the edges represent the degree of the interaction between these nodes. FCMs have several appealing characteristics in modeling the complex relationship between variables. One is that FCM is flexible and easy to be constructed. Second, FCM has powerful ability to handle uncertainties. Finally, FCM can be sufficiently trained with small samples. With these advantages, FCMs have been widely used to solve different scientific and engineering problems, for example, in [9], an enhanced FCM called E-FCMs is proposed to model the influence strength between brain activity associated with different regions of the brain. In [10], the FCM is applied to gene regulatory network reconstruction, and a preference-based evolutionary bi-objective algorithm was developed to lean the FCM. In [11], the FCM was used to predict the DNA-Binding residues in local segments of protein sequences. In [12], FCM was used to model the discontinuous behavior in a complex system. In [13], a higher-order FCM combined with sparse auto-encoder was proposed for time series predicting and a batch gradient descent method is adopted to learn the FCM. In [14], a fast and effective method based on constrained convex optimization was developed for FCM learning. In [15], FCM was used to control wheeled mobile robot.

Although FCMs have found successful applications in many fields, they still suffer from several drawbacks. First, FCMs adopt the sigmoid function as the nonlinear transfer function. Recent deep learning researches have revealed that the sigmoid function can lead to poor performance due to its saturation near 0 and 1, which limits its ability to learn nonlinear mappings. Second, gradient-based methods are traditionally used as the learning algorithms for FCMs. Gradient-based methods are sensitive to noise and can easily be trapped in a local optimum. Recently, several population-based methods have been proposed to learn FCMs due to their global searching characteristics. In [16], the real-coded genetic algorithm (RCGA) was used to learn FCMs, where the weights of FCMs to be learned were encoded as an individual of GA. Compared to GA, particle swarm optimization (PSO) has simpler evolutionary operations. Therefore, in [17], PSO was adopted to learn FCMs. The differential evolution (DE) [18], the asexual reproduction optimization (ARO) algorithm [19], and the imperialist competition algorithm (ICA) [20] have also been adopted to learn FCMs.

ICA is an effective evolutionary algorithm first proposed by Atashpaz-Gargari and his colleagues [21]. It shows great potential in solving different complex optimization problems, such as flexible job shop scheduling [22], neural network training [23], parameter optimal design of heat exchangers [24], controller parameter tuning [25], and node placement in wireless sensor networks [26]. However, like many other evolutionary algorithms, ICA is also plagued by the premature problem. That is to say, if ICA finds a local optimal solution and fails to jump out of it, ICA will converge to the local optimum, which is undesired. To enhance the search ability of ICA, several ICA variants have been proposed. These variants can be roughly classified into three classes. The first class is to change the assimilation strategy. For example, in [27], an ICA variant called AR-ICA was proposed, where the assimilation coefficients are dynamically adjusted based on the concept of attraction and repulsion to better balance the diversity and convergence of the algorithm. In [28, 29], a modified ICA was proposed, in which the colonies move to its imperialist using a velocity-position model like PSO algorithm. In [30], a bare-bone ICA (BB-ICA) was given. In BB-ICA, the new position of each colony is determined according to a Gaussian sampling. In [31], an ICA variant called Gbest-ICA was proposed via taking the difference between the colony’s current position and the global best position into account when colony moves towards its imperialist. An orthogonal ICA (O-ICA) was proposed in [32], where a new move direction orthogonal to the original move direction is added to the colonies’ movement formula. The other class of ICA variants is to modify the original ICA’s competition and revolution step. In [33], the chaotic map was used to generate the revolted colonies, while in [34], the GA mutation operator is used to generate new revolted colonies to replace some original colonies. In [35], a clone evolution operator is introduced into the competition phase. Apart from the above two classes of variants, some researchers hybrid ICA with other evolutionary algorithms to improve the performance of ICA. For instance, in [36], the hybrids of ICA and simplex method was proposed. In the hybrid algorithm, the simplex method is responsible for a local searching. In [37], the ICA is hybridized with simulating annealing (SA), in which the SA is used to improve the position of imperialist after assimilation step. In [38], ICA is mixed with pattern search algorithm, the results of ICA severing as initial position are further improved using pattern search.

With the above facts in mind, we develop in this paper an improved FCM in which two improvements are made. First, a ReLU function is adopted to replace the original sigmoid function to enhance the nonlinear mapping ability. Second, an improved ICA, which integrates the quasi-opposition learning and bare bone ICA, is proposed to learn FCMs’ weights. The proposed method is used to predict the employment rate of university graduates.

The rest of this paper is arranged as follows. Some basics about FCMs are provided in Section 2. The bare-bone imperialist competitive algorithm is detailed in Section 3. The proposed method for predicting the employment rate of graduates is presented in Section 4. The prediction results are shown in Section 4.4. Finally, the concluding remarks are given in Section 5.2.

## 2 ReLU-FCM

**Fig 1:**
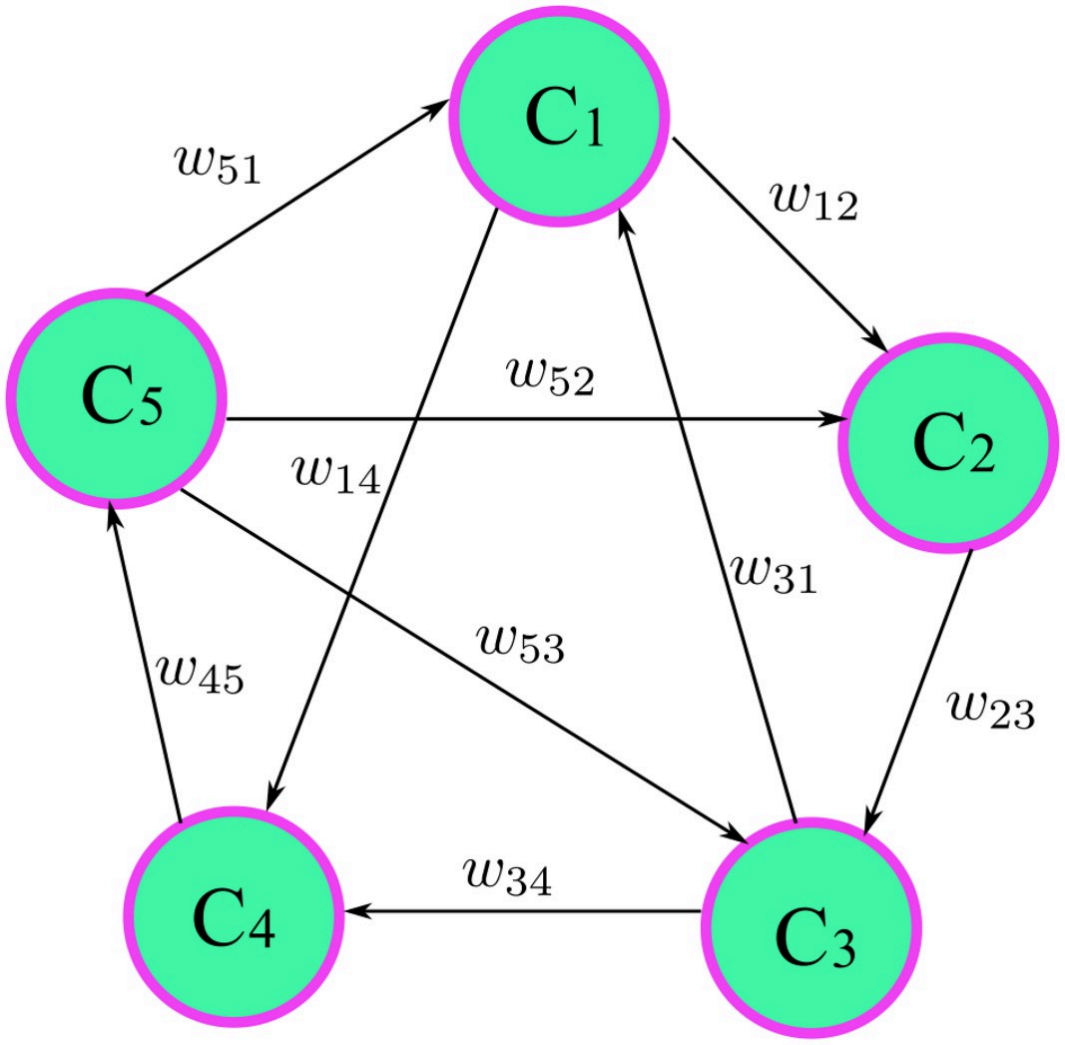
The structure of FCM.

FCMs, as shown in Fig 1, can be viewed as a fuzzy version of Axelrod’s cognitive maps. The causality between the nodes in an FCM can be a fuzzy degree. Like cognitive maps, an FCM consists of a set of nodes and weighted edges. The nodes represent the concepts or variables of a system while the weighted edges denote the causality between these concepts or variables. Suppose there are *n* nodes in an FCM, denoted as {*C*_1_, *C*_2_, ⋯, *C*_*n*_}. The weight of the edge from node *C*_*j*_ to *C*_*i*_ is denoted by *w*_*ji*_, where *i*, *j* = 1, 2, ⋯, *n*. The value of *w*_*ji*_ lies in [−1, 1]. The value of weight *w*_*ji*_ explains the degree of the causality between the nodes, and the sign of weight *w*_*ji*_ denotes the influential direction of the connected nodes. Specifically, if *w*_*ji*_ > 0, it means that an increase of node *C*_*j*_ results in an increase of node *C*_*i*_ with strength *w*_*ji*_ and vice versa. If *w*_*ji*_ < 0, it means an increase of node *C*_*j*_ results in a decrease of node *C*_*i*_ with strength |*w*_*ji*_| and vice versa. If *w*_*ji*_ = 0 means there is no relation between node *C*_*j*_ and node *C*_*i*_, the weights of all edges in an FCM can be rewritten into a matrix form as follows:

$$\begin{array}{c} W=[\begin{array}{cccc} w_{11} & w_{12} & \cdots & w_{1n}\\ w_{21} & w_{22} & \cdots & w_{2n}\\ \vdots & \vdots & \ddots & \vdots \\ w_{n1} & w_{n2} & \cdots & w_{nn} \end{array}]=[\begin{array}{cccc} w_{1} & w_{2} & \cdots & w_{n} \end{array}] \end{array}$$ (1)

In the FCM, each node at time *t* has a value, called activation or state value, denoted by *a*_*i*_(*t*). The state values of nodes vary with time. At time *t* + 1, the state value is calculated as:

$$\begin{array}{c} a_{i}\left(t+1\right)=f(\sum_{j=1}^{n}a_{j}\left(t\right)w_{ji}),i=1,2,\cdots ,n, \end{array}$$ (2)

where *f*(⋅) is the transfer function or activation function. Usually, the sigmoid function can be expressed as:

$$\begin{array}{c} f\left(x\right)=\frac{1}{1+e^{-\lambda x}} \end{array}$$ (3)

is selected as the transfer function. As stated before, the sigmoid function has several drawbacks that limit its nonlinear mapping performance. In this paper, the ReLU function is adopted to replace the sigmoid function. The ReLU function is defined as:

$$\begin{array}{c} f(x)=\text{max}(0,x). \end{array}$$ (4)

Denote **a**(*t*) = [*a*_1_(*t*), *a*_2_(*t*), ⋯, *a*_*n*_(*t*)]^T^. Then (2) can be written into the following form:

$$\begin{array}{c} a_{i}\left(t+1\right)=f\left(a\left(t\right)w_{i}\right). \end{array}$$ (5)

In (5), the state value of each node at time *t* + 1 depends on the state values of all nodes at time *t* only. If the state value of each node at time *t* + 1 depends on the state values of all nodes at both time *t* and time *t* − 1, ⋯, *t* − *k* + 1, i.e.,

$$\begin{array}{c} a_{i}\left(t+1\right)=f\left(a\left(t\right)w_{i}^{1}+a\left(t-1\right)w_{i}^{2}+\cdots +a\left(t-k+1\right)w_{i}^{k}\right), \end{array}$$ (6)

then, the FCM is called a high-order FCM. Eq (6) can be written in a matrix form as follows:

$$\begin{array}{c} a_{i}\left(t+1\right)=f(\sum_{j=1}^{k}a\left(t-j+1\right)w_{i}^{j}), \end{array}$$ (7)

To enable the FCM to describe the complex dynamics of a system, we must determine its weights first. The weights can be determined in two ways, by using expert knowledge and by learning from actual data. In this paper, the weights of the FCM are obtained by learning. An improved ICA called BICA is developed to perform the learning.

## 3 Quasi-oppositional bare-bone imperialist competitive algorithm

### 3.1 Quasi-opposition learning

Opposition-based learning (OBL) was first introduced by Tizhoosh in 2005 [39] inspired by the Ying-Yang concept. OBL aims to find better solutions for machine learning tasks in an opposition direction. Since its introduction, OBL has been applied in several different fields such as neural network training and evolutionary algorithm construction [39]. OBL can be used to improve the performance of original methods. However, the opposition points frequently exceed the searching range and are therefore unable to produce higher performance. To mitigate this problem, in this paper, we focus on a variant of OBL, i.e., quasi-opposition based learning (QOBL). The related concepts are stated as follows.

**Definition 1** (*Opposite number*) Let *x* ∈ [*a*, *b*] be a real number. Its opposite $\overset{˘}{x}$ is defined as:

$$\begin{array}{c} \overset{˘}{x}=a+b-x. \end{array}$$ (8)

**Definition 2** (*Opposite point in the D-dimensional space*) [39] Let **x** = [*x*_1_, *x*_2_, ⋯, *x*_*D*_] be a point in *D*-dimensional space and *x*_*i*_ ∈ [*a*_*i*_, *b*_*i*_], *i* = 1, 2, ⋯, *D*. The opposition of **x** is denoted as $\overset{˘}{x}=\left[{\overset{˘}{x}}_{1},{\overset{˘}{x}}_{2},\cdots ,{\overset{˘}{x}}_{D}\right]$, where each element of $\overset{˘}{x}$ is given as:

$$\begin{array}{c} {\overset{˘}{x}}_{i}=a_{i}+b_{i}-x_{i}. \end{array}$$ (9)

**Definition 3** (*Quasi-opposite number*) Let *x* ∈ [*a*, *b*] be a real number. Its quasi-opposite number $\bar{x}$ is defined as:

$$\begin{array}{c} \bar{x}=\{\begin{array}{cc} \frac{a+b}{2}+r\left(\overset{˘}{x}-\frac{a+b}{2}\right), & \overset{˘}{x}<\frac{a+b}{2};\\ \overset{˘}{x}+\left(\frac{a+b}{2}-\overset{˘}{x}\right), & \overset{˘}{x}\geq \frac{a+b}{2}. \end{array} \end{array}$$ (10)

**Definition 4** (*Quasi-opposite point in the D-dimensional space*) Let **x** = [*x*_1_, *x*_2_, ⋯, *x*_*D*_] be a point in *D*-dimensional space and *x*_*i*_ ∈ [*a*_*i*_, *b*_*i*_], *i* = 1, 2, ⋯, *D*. The quasi-opposition of **x** is denoted as $\bar{x}=\left[{\bar{x}}_{1},{\bar{x}}_{2},\cdots ,{\bar{x}}_{D}\right]$, where each element of $\bar{x}$ is given as:

$$\begin{array}{c} {\bar{x}}_{i}=\{\begin{array}{cc} \frac{a+b}{2}+r\left({\overset{˘}{x}}_{i}-\frac{a+b}{2}\right), & {\overset{˘}{x}}_{i}<\frac{a+b}{2};\\ {\overset{˘}{x}}_{i}+\left(\frac{a+b}{2}-{\overset{˘}{x}}_{i}\right), & {\overset{˘}{x}}_{i}\geq \frac{a+b}{2}. \end{array} \end{array}$$ (11)

**Definition 5** (Opposition or quasi-opposition based optimization) Let **x** = (*x*_1_, *x*_2_, ⋯, *x*_*D*_) be a point in *D*-dimensional space and *f*(**x**) the fitness function. The opposition and quasi-opposition point of **x** is denoted as $\overset{˘}{x}$ and $\bar{x}$, respectively. If $f(\overset{˘}{x})$ or $f(\bar{x})$ is better than *f*(**x**), then point **x** is replaced with $\overset{˘}{x}$ or $\bar{x}$; otherwise, point **x** is reserved.

### 3.2 Bare bone ICA (BBICA)

BICA involves the same main steps as basic ICA, including initialization, assimilation, imperialistic competition, and convergence. These steps are described in detail in the following sections.

#### 3.2.1 Initialization

In this step, the main task is to generate initial countries, and then classify them into imperialists and colonies according to their power. Furthermore, the colonies are probably assigned to each imperialist to form empires. Suppose a country is represented by a vector **x**_*i*_ = [*x*_*i*1_, *x*_*i*2_, ⋯, *x*_*iD*_] and each element *x*_*ij*_ of vector **x**_*i*_ lies in [*l*_*j*_, *u*_*j*_], which is randomly generated as:

$$\begin{array}{c} x_{ij}=l_{j}+rand\times \left(u_{j}-l_{j}\right),i=1,2,\cdots ,N,j=1,2,\cdots ,D, \end{array}$$ (12)

where *rand* is a random number distributed in [0, 1]. To evaluate each country, the cost of each country is calculated as $c_{i}=F\left(x_{i}\right)$, where F(·) is called the cost function. Once *N* countries are generated, they will be classified into imperialists and colonies according to their power, i.e., the cost of each country. Usually, the first *N*_*imp*_ countries are selected as the imperialists and the remaining *N*_*col*_ = *N* − *N*_*imp*_ countries are classified as colonies. Then, the colonies are assigned to each imperialist. The *n*-th imperialist occupies *NC*_*n*_ colonies, which are calculated as:

$$\begin{array}{c} NC_{n}=round\left\{p_{n}\cdot N_{col}\right\}, \end{array}$$ (13)

where

$$\begin{array}{c} p_{n}=|\frac{c_{n}-{\text{max}}_{i=1,2,\cdots ,N\{c_{i}\}}}{\sum_{i=1}^{N_{imp}}\left(c_{n}-{\text{max}}_{i=1,2,\cdots ,N}\left\{c_{i}\right\}\right)}| \end{array}$$ (14)

is the normalized power of an imperialist and *c*_*n*_ is the cost of the *n*-th imperialist. The *NC*_*n*_ colonies together with the imperialist make up the *n*-th empire.

#### 3.2.2 Assimilation

**Fig 2:**
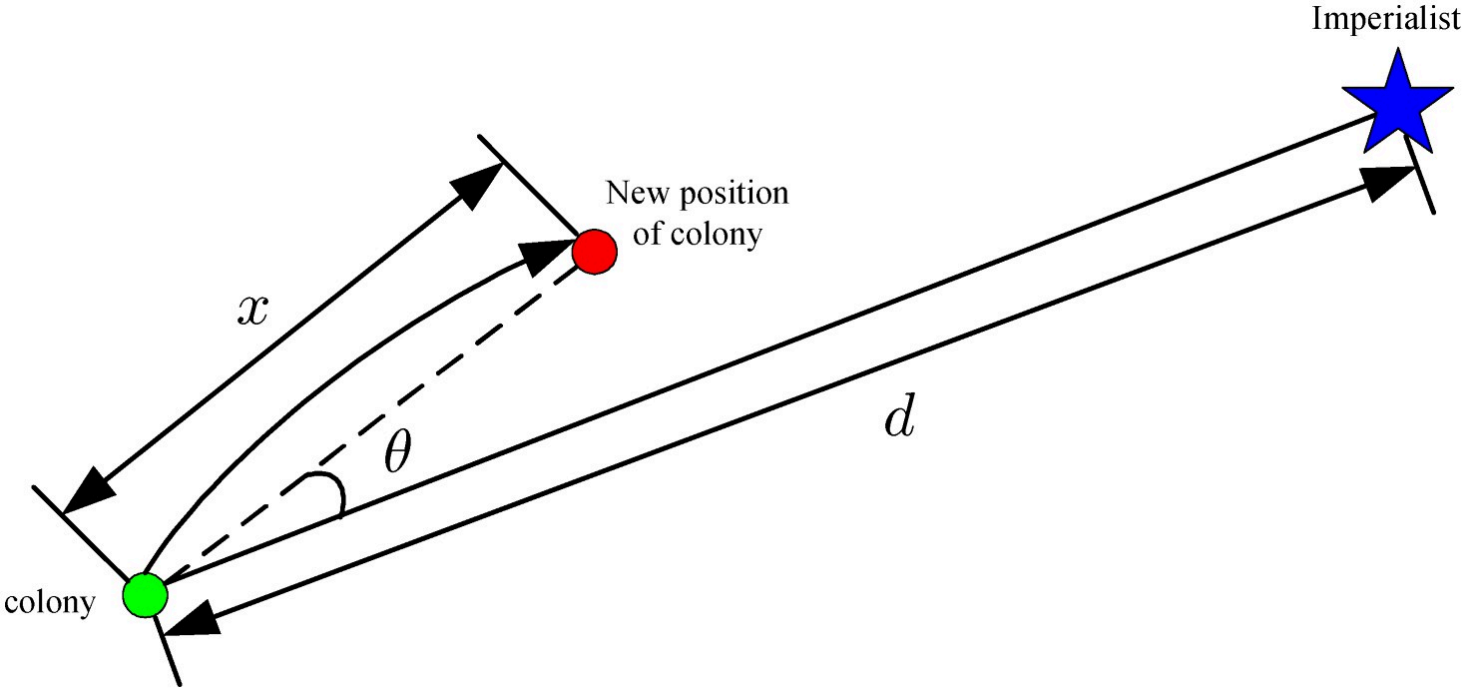
Movement of colonies towards imperialists in the ICA.

After initialization, the colonies in each empire move towards the corresponding imperialists to increase the total power of the empire. This process is called the assimilation step. In the original ICA, the colonies move towards their corresponding imperialists as shown in Fig 2. However, the assimilation is an important step, which is responsible for the exploitation and exploration of the ICA. A good strategy should strike a good balance between exploration and exploitation. To this end, a Gaussian sampling technique is adopted as the assimilation strategy in the BICA. Specifically, the new position of a colony is determined as:

$$\begin{array}{c} x_{i,j}^{\prime }=N\left(\mu_{j},\sigma_{j}\right), \end{array}$$ (15)

where $x_{i,j}^{\prime }$ represents the *j*-th variable of $x_{i}^{\prime }$, i.e., the new position of the *i*-th colonies in an empire, and *N*(*μ*_*j*_, *σ*_*j*_) is a Gaussian distribution random number with mean *μ*_*j*_ and variance *σ*_*j*_. The mean and variance are calculated as:

$$\begin{array}{c} \mu_{j}=\left(x_{i,j}+x_{j}^{imp}\right)/2 \end{array}$$ (16)

and

$$\begin{array}{c} \sigma_{j}=\left|x_{j}^{imp}-x_{i,j}\right|, \end{array}$$ (17)

where $x_{j}^{imp}$ is the *j*-th variable of the imperialist position.

#### 3.2.3 Imperialistic competition

After assimilation, the power of each empire is enhanced. Then, the competition among empires begins. The competition leads more powerful empires to possess more colonies and the weaker ones to lose their colonies. The lost colonies from the weaker empires are reassigned to other more powerful empires via competition. Let the *ℓ*-th empire be the weakest. The probability of the *n*-th empire to possess the colonies is given as:

$$\begin{array}{c} p_{n}=|\frac{NTC_{n}}{\sum_{i=1}^{N_{imp}}NTC_{i}}|, \end{array}$$ (18)

where *NTC*_*n*_ = *TC*_*n*_ − max_*i*_{*TC*_*i*_}, *i* = 1, 2, ⋯, *N*_*imp*_ and *TC*_*i*_ is the total cost of the *i*-th empire. Define a probability vector *P* as

$$\begin{array}{c} P=[p_{1},p_{2},\cdots ,p_{N_{imp}}], \end{array}$$ (19)

and a probability vector *R* as

$$\begin{array}{c} R=[r_{1},r_{2},\cdots ,r_{N_{imp}}],r_{i}∼U\left(0,1\right) \end{array}$$ (20)

The difference vector between *P* and *R* is

$$\begin{array}{c} D=P-R. \end{array}$$ (21)

The empire whose relevant index is maximum in *D* will occupy the weakest colony of the weakest empire.

#### 3.2.4 Convergence

After empire competition, all the empires will collapse, except the most powerful one, and all the colonies will belong to this empire. As a consequence, all the colonies along with the unique imperialist will be located in the same position and have the same cost. In such a condition, the competition is stopped and the algorithm is terminated.

### 3.3 Quasi-oppositional bare-bone ICA (QBBICA)

To further improve the global searching performance of BBICA, in this paper, quasi-opposition learning is combined with BBICA. The resulting algorithm is called QBBICA. The difference between BBICA and QBBICA lies in the initialization and assimilation steps.

#### 3.3.1 Quasi-oppositional initialization

In QBBICA, after randomly generating *N* countries according to Eq (12), another *N* corresponding quasi-oppositional countries are generated according to the concept of quasi-oppositional point (11). Then, we calculate the fitness values of the 2*N* countries and sort them in an ascending order. The first *N* countries are selected as the initial population.

#### 3.3.2 Quasi-oppositional assimilation

In QBBICA, after all the colonies in an empire have moved towards their corresponding imperialists according to Eq (15), a set of quasi-oppositional colonies are generated according to Eq (11). Similarly, we calculate the fitness values of new colonies and their corresponding quasi-oppositional colonies, and sort the values in an ascending order. The first *N* colonies are selected the final colonies in the empire. For complex optimization problems with many local optimum, exploring the information of oppositional point of current location may provide more help to find global optimum. The reasons are as follows. First, the oppositional point maybe result in a better function value, and can speed up the convergence of ICA algorithm. Second, the oppositional direction of current location maybe provide a better descent direction and thus has a large probability to escape the local optimal. Therefore, the oppositional operator can greatly improve the performance of ICA algorithm.

#### 3.3.3 Computation complexity of QBBICA

QBBICA mainly consists of four steps, i.e., initialization, assimilation, revolution and imperialistic competition. For a *D* dimension problem and QBBICA with *N* countries and the maximum iteration number *iter*_*max*_, the computation complexity for generating *N* initial countries and evaluate its fitness is *O*(*N* * *D* + *N* * *nF*), where *nF* is a number of function evaluation. The computation complexity of generating *N* quasi-oppositional countries and fitness evaluation is *O*(*N* * *D* + *N* * *nF*). Therefore, in the initialization step, the computation complexity of QBBICA is 2*O*(*N* * *D* + *N* * *nF*). In the iterative process, the computation complexity of assimilation step is, similarly, 2*O*(*iter*_*max*_ * (*N* * *D* + *N* * *nF*)). The computation complexity of revolution step is *O*(*iter*_*max*_ * (*rr* * *N* + *nF* * *rr* * *N*) and that of imperialist competitive is *O*(*iter*_*max*_ * (*N*_*imp*_ + *N*_*imp*_ * *N*_*col*_)), where *rr* is the revolution rate and *N*_*imp*_ the number of imperialist, *N*_*col*_ the number of colonies. Finally, the computation complexity of QBBICA is *O*(*iter*_*max*_ * *N* * *D* * *nF*).

## 4 Predicting employment rate of graduates using proposed method

In this section, we describe in detail how our proposed method is used to predict the employment rate of graduates. Inspired by the work of Wojciech [40], we divide the prediction process into five steps. First, the historical employment rate series is transformed into a fuzzy time series (FTS). Second, the FTS is used to construct an FCM with the ReLU activation function. Third, the FCM is trained using QBBICA. Then, the trained FCM evolves into one or more steps to obtain a predicted fuzzy state. Finally, the predicted fuzzy state is defuzzified to obtain the numerical predicted values of employment rate.

### 4.1 Fuzzy time series

Let *Y* = {*y*(*t*), *t* = 1, 2, ⋯, *n*} be the historical time series of employment rate, and $\mu_{A_{i}}:R\to \left[0,1\right],i=1,2,\cdots ,m$ a set of membership functions. The membership value of each *y*(*t*) belonging to fuzzy set $A_{i}\in \tilde{A}$ is evaluated using $\mu_{A_{i}}$. Thus, the original time series *y*(*t*) is transformed into FTS *F*(*t*) = {*f*_1_(*t*), *f*_2_(*t*), ⋯, *f*_*m*_(*t*)}. In this paper, the triangle membership function is adopted.

### 4.2 Construction of FCM

After the original time series *y*(*t*) is transformed into FTS *F*(*t*), an FCM is constructed. At any time *t*, there are *m* membership values *f*_1_(*t*), *f*_2_(*t*), ⋯, *f*_*m*_(*t*) corresponding to *y*(*t*). The *m* membership values represent the state of the FCM at time *t*. Therefore, an FCM with *m* nodes is constructed, and the activation value of the *i*-th node equals to the membership value *f*_*i*_(*t*) at time *t*.

### 4.3 Training of FCM using QBBICA

After the FCM is constructed, the weights of FCM should be determined through training. In the literature, many learning algorithms have been developed for FCM learning, such as Hebbian-based, population-based and hybrid algorithms. See [41] for more details. In this paper, the proposed QBBICA is used to learn the FCM. The main steps are illustrated as follows.

**Step 1** Preparing employment rate data {*y*(*t*), *t* = 1, 2, ⋯, *n*}. The data is normalized into [0, 1]. The first 60% is used as training samples and the remaining as test samples. The employment rate data is normalized and then transformed into *m* fuzzy sets with the triangle membership function. Consequently, the *m* FTS *f*_*i*_(*t*) is obtained.
**Step 2** Constructing the FCM. According to the order *k* of the FCM and the node number *m*, an FCM with *m* nodes is constructed. The status value of FCM at time *t* is *f*_1_(*t*), *f*_2_(*t*), ⋯, *f*_*m*_(*t*).
**Step 3** Determining the algorithm parameters of QBBICA such as the number of countries, the number of empires, and the maximum number of iterations.
**Step 4** Solution representation. If the FCM has *m* nodes, then each weight matrix is a *m* × *m* matrix. Furthermore, if the order is *k*, then there are *k* weight matrices. Therefore, a vector with length *k* × *m* × *m* is used to represent each country in QBBICA.
**Step 5** Initialization. In the FCM, each weight lies in [−1, 1]. In this step, *N* vectors with length *k* × *m* × *m* in [−1, 1] are randomly generated. The *r*-th country is denoted as **x**_*r*_.
**Step 6** Calculating the fitness of each country. To evaluate the power of each country, their fitness is calculated. First, each vector **x**_*r*_ is reshaped into *k* matrices with size *m* × *m*, which is denoted as $w_{i}^{j}$. Then, the fitness of each country **x**_*r*_ is defined as:
  $$\begin{array}{c} F\left(x_{r}\right)=\frac{1}{ml}\sum_{t=1}^{l}\sum_{j=1}^{k}\sum_{i=1}^{m}{\left(a_{i}\left(t\right)-{\hat{a}}_{i}\left(t\right)\right)}^{2}, \end{array}$$ (22)
  where
  $${\hat{a}}_{i}\left(t\right)=f(\sum_{j=1}^{k}a\left(t-j+1\right){\hat{w}}_{i}^{j})$$
  is the estimated state and *a*_*i*_(*t*) is the target state.
**Step 7** According the fitness values of the countries, the whole countries are classified into colonies and imperialists and assigned to their corresponding imperialists to form empires.
**Step 8** Perform assimilation on each empire as described in subsection 3.2.2.
**Step 9** Perform empire competition as described in subsection 3.2.3.
**Step 10** Repeat Step 7 and Step 8 until the convergence condition is met.
**Step 11** Output the best vector with the minimum fitness value. Reshaping the vector into weight matrices.

### 4.4 Predicting employment rate using FCM

After the FCM is trained, it is used to predict the employment rate. Suppose that the state value of the FCM at time *t* is {*a*_1_(*t*), *a*_2_(*t*), ⋯, *a*_*m*_(*t*)}, where each *a*_*i*_(*t*) represents the degree of *y*(*t*) belonging to the *i*-th fuzzy set, it is a linguistic variable. After the weights of the FCM are determined, the FCM evolves into a step according to Eq (7) to obtain the predicted state values at *t* + 1, i.e., ${\hat{a}}_{i}\left(t+1\right)$. The linguistic value ${\hat{a}}_{i}\left(t+1\right)$ is transformed into a numerical value using the defuzzification technique. The predicted numerical value $\hat{y}\left(t+1\right)$ is obtained as:

$$\begin{array}{c} \hat{y}\left(t+1\right)=\frac{\sum_{i=1}^{m}{\hat{a}}_{i}\left(t+1\right)mp_{i}}{\sum_{i=1}^{m}{\hat{a}}_{i}\left(t+1\right)}, \end{array}$$ (23)

where *mp*_*i*_ is the center of the *i*-th fuzzy set.

## 5 Results

### 5.1 Performance of QBBICA

Here, the optimization performance of the proposed QBBICA is validated using several benchmark functions. The selected benchmark functions are listed in Table 1. In those six functions, the Sphere function is unimodal and the others are multimodal. To show the advantage of the proposed QBBICA, we compare it with four other algorithms, i.e., GA, PSO, ICA, and BBICA. The parameter settings of these algorithms are as follows. For GA, the “tournament” selection, the “polynomial mutation” and the “Simulated Binary Crossover (SBX)” are adopted. For PSO, *c*_1_ = *c*_2_ = 2, and the inertia weight decreases linearly from *w*_max_ = 0.9 to *w*_min_ = 0.4. For DE, the crossover rate CR = 0.8, mutation factor F = 0.8, and “DE / rand-to-best / 1 / bin” strategy is adopted. The parameters for ICA, BBICA, and the proposed QBBICA are set as in [21], which are set as follows, the assimilation coefficient *γ* = 2, revolution rate equals to 0.3, the number of initial imperialist is 8. For each algorithm, the population size is 50 and the maximum number of iterations is 1000. All the algorithms independently run 30 times.

**Table 1:** Benchmark functions.

No.	Function name	Expression	Bounds	Global minimum
*f*_1_	Sphere	$f\left(x\right)=\sum_{i=1}^{D}x_{i}^{2}$	[-5.12, 5.12]	*f*(x*) = 0
*f*_2_	Rastrigin	$f\left(x\right)=10D+\sum_{i=1}^{D}\left(x_{i}^{2}-10\;\text{cos}\left(2\pi x_{i}\right)\right)$	[-5.12, 5.12]	*f*(x*) = 0
*f*_3_	Griewank	$f\left(x\right)=\sum_{i=1}^{D}\frac{x_{i}^{2}}{4000}-\prod_{i=1}^{D}\;\text{cos}(\frac{x_{i}}{\sqrt{i}})+1$	[-600, 600]	*f*(x*) = 0
*f*_4_	Ackley	$f\left(x\right)=-20\;\text{exp}(-0.2\sqrt{\frac{\sum_{i=1}^{D}x_{i}^{2}}{D}})-\text{exp}(\frac{\sum_{i=1}^{D}\text{cos}\left(2\pi \cdot x_{i}\right)}{D})+20+e$	[-32,32]	*f*(x*) = 0
*f*_5_	Alpine	$f\left(x\right)=\sum_{i=1}^{D}\left|x_{i}\;\text{sin}\left(x_{i}\right)+0.1x_{i}\right|$	[-10,10]	*f*(x*) = 0
*f*_6_	Pathologic	$f\left(x\right)=\sum_{i=1}^{D-1}{\left(0.5+\frac{{\text{sin}}^{2}(\sqrt{100x_{i}^{2}+x_{i+1}^{2}})-0.5}{1+0.001(x_{i}^{2}-2x_{i}x_{i+1}+x_{i+1}^{2})}\right)}^{2}$	[-100,100]	*f*(x*) = 0

**Fig 3:**
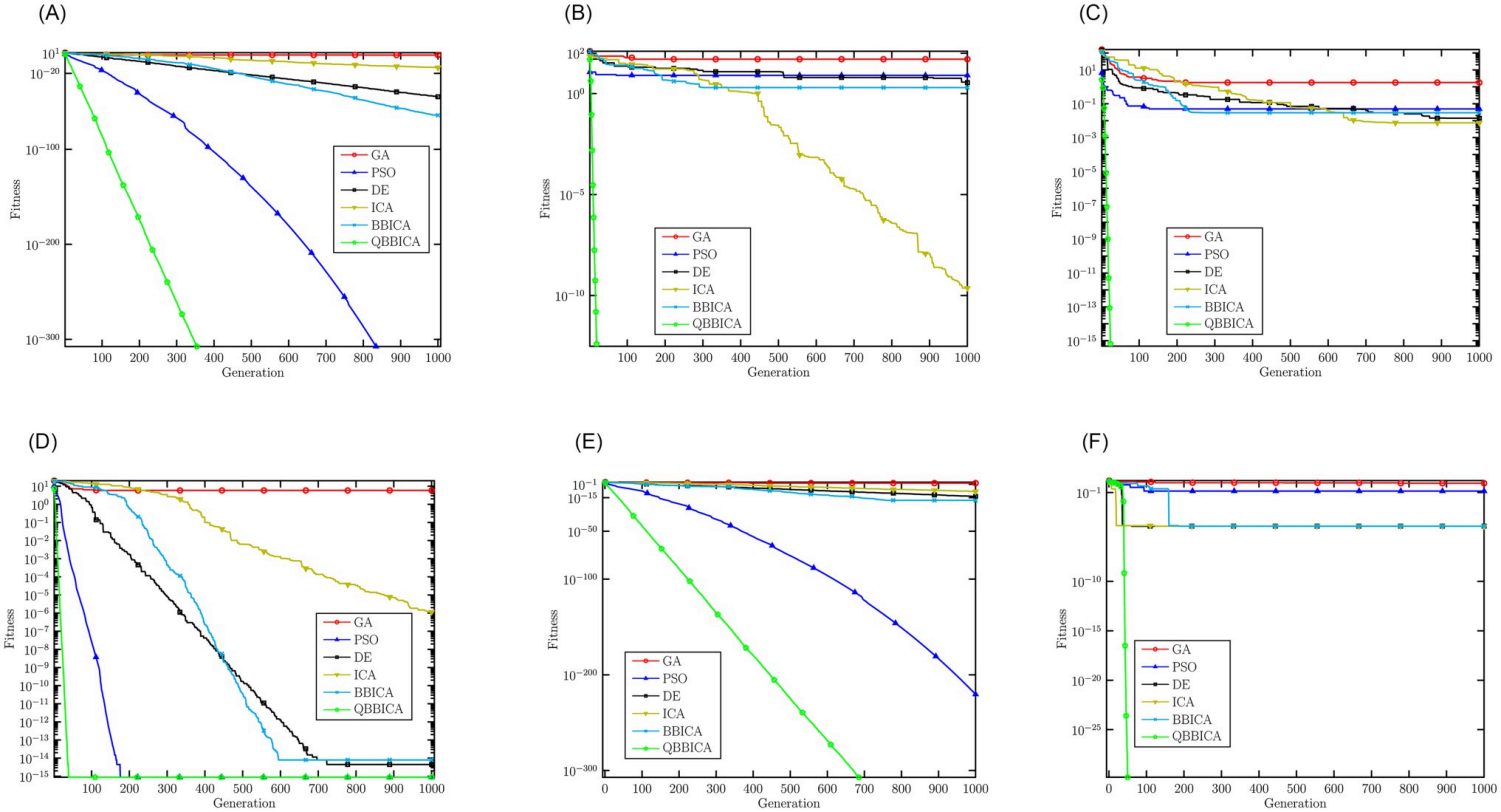
Convergence curves of different algorithms on the benchmark functions. (a) Sphere, (b) Rastrigin, (c) Griewangk, (d) Ackley, (e) Alpine, (f) Pathologic.

Table 2 shows the optimization results of benchmark functions with 10 dimension obtained by each algorithm, including the best, the worst and the mean fitness. It can be seen that our proposed QBBICA achieves better optimization results than the other four algorithms. To compare the convergence speeds of these algorithms, Fig 3 shows the fitness convergence curves of the algorithms. It is clear that our proposed QBBICA has a faster convergence speed than the other algorithms. To sum up, our proposed QBBICA not only has a stronger global search ability but also a faster convergence speed than the other algorithms. To compare the superiority of the proposed QBBICA, a Wilcoxon sign-rank-sum test had been conducted and the *h* values of the test are listed in Table 3. The *h*-value equaling to 1 indicates that QBBICA is better than other algorithm. From Table 3, one can see that the proposed QBBICA performs better than other algorithms except for PSO on sphere function. Furthermore, to demonstrate the computational efficiency, the average computation time of each algorithm during the 30 runs for each function are listed in Table 4.

**Table 2:** Results of benchmark function optimization.

		GA	PSO	DE	ICA	BBICA	QBBICA
Sphere	Best	0.1910	0	2.0074E-45	1.0428E-14	7.4827E-65	0
	Worst	0.1910	0	1.1405E-43	7.9616E-11	1.6881E-56	0
	Mean	0.1910	0	2.3425E-44	4.6654E-12	5.9441E-58	0
Rastrigin	Best	50.5737	8.0129	3.4928	2.3120E-10	1.9899	0
	Worst	50.5737	11.4694	9.3382	0.9950	14.9244	0
	Mean	50.5737	10.0255	6.4821	0.0664	5.9366	0
Griewangk	Best	1.7835	0.0495	0.0140	0.0074	0.0296	0
	Worst	1.7835	0.7751	0.1607	0.1205	0.3545	0
	Mean	1.7835	0.4382	0.0825	0.0658	0.1233	0
Ackley	Best	5.8070	8.8818E-16	4.4409E-15	1.15E-06	7.9936E-15	8.8818E-16
	Worst	5.8070	7.9936E-15	4.4409E-15	0.0001	5.0626E-14	8.8818E-16
	Mean	5.8070	4.0856E-15	4.4409E-15	1.8754E-05	1.6875E-14	8.8818E-16
Alpine	Best	1.9266	3.7499E-221	2.57E-14	2.8744E-09	1.7347E-18	0
	Worst	1.9266	0.0082	0.0006	1.1307E-05	4.8017E-14	0
	Mean	1.9266	0.0021	4.8514E-05	1.1562E-06	8.2664E-15	0
Pathologic	Best	0.8563	0.1374	3.8916E-05	3.8916E-05	3.8916E-05	0
	Worst	0.8563	0.9631	0.7140	0.4760	0.6371	0
	Mean	0.8563	0.3071	0.1984	0.1190	0.1706	0

**Table 3:** *h*-value of Wilcoxon test results.

QBBICA vs GA	1	1	1	1	1	1
QBBICA vs PSO	0	1	1	1	1	1
QBBICA vs DE	1	1	1	1	1	1
QBBICA vs ICA	1	1	1	1	1	1
QBBICA vs BBICA	1	1	1	1	1	1

**Table 4:** Average CPU time (s) of all the referenced algorithm on benchmark function.

	GA	PSO	DE	ICA	BBICA	QBBICA
Sphere	0.9580	0.8255	0.2089	0.4841	0.4400	0.3564
Rastrigin	1.2230	0.9875	0.2620	0.6144	0.5915	0.3790
Griewangk	1.5901	1.3662	0.2739	0.5269	0.4867	0.3859
Ackley	1.4963	1.2712	0.2715	0.5843	0.5382	0.4450
Alpine	1.5689	1.3050	0.2640	0.6727	0.7381	0.4555
Pathologic	1.8525	1.5785	0.2842	0.7143	0.6690	0.4965

### 5.2 Predicting employment rate

In this section, experiments are conducted to predict the employment rate of graduates from Liren College, Yanshan University. The employment rate is the ratio of the number of the employed graduates to the total number of graduates. The employment rate data of Liren College from 2005 to 2020 is used in the experiments, which is listed in Table 5.

**Table 5:** Employment rate data of Liren College, Yanshan University from 2005 to 2020.

Year	2005	2006	2007	2008	2009	2010	2011	2012
E-rate	84.68	86.54	88.32	90.44	88.11	90.21	88.93	90.59
Year	2013	2014	2015	2016	2017	2018	2019	2020
E-rate	88.59	89.93	90.19	90.19	91.07	90.83	90.37	95.31

The first 60% of the employment rate data is used as the training sample and the remaining 40% as the test sample.

In the experiments, five triangle membership functions are adopted to transform the employment rate into an FTS. Thus, there are five nodes in the FCM. The order of the FCM is set to 2 in this paper. Thus, there are two weight matrices and the size of each matrix is 5 × 5. For the purpose of comparison, the RCGA, PSO, ICA, BBICA and the proposed QBBICA are implemented and used to train the **ReLU-FCM** and the original FCM, denoted as **Sig-FCM**. For the sake of convenience, the FCMs trained by these algorithms are denoted as RCGA-FCM, PSO-FCM, ICA-FCM, BICA-FCM, and QBBICA-FCM, respectively. In these algorithms, each individual is represented by a vector with length 50. Each algorithm has 200 individuals, and the maximum number of iterations of each algorithm is 1000. In addition, to reveal its effectiveness in employment rate prediction, the FCM based prediction methods are also compared back propagation neural network, linear regression model and grey GM(1,1) model.

To objectively reflect the performance of the FCMs trained by the different algorithms, root mean square error (RMSE), coefficient of determination *R*^2^, coefficient of efficiency *E*_*sn*_, and mean absolute error (MAE) are used as the indices for performance evaluation. They are defined as follows:

$$\begin{array}{c} \text{RMSE}=\sqrt{\frac{\sum_{i=1}^{N}{\left(y_{i}-{\hat{y}}_{i}\right)}^{2}}{N}}, \end{array}$$ (24)

where *y*_*i*_ and ${\hat{y}}_{i}$ are the real and predicted values, respectively, and *N* is the total number of data points used in the experiment.

Coefficient of determination:

$$\begin{array}{c} R^{2}=[\frac{\sum_{i=1}^{N}\left(y_{i}-\bar{y}\right)\left({\hat{y}}_{i}-\bar{\hat{y}}\right)}{\sqrt{\sum_{i=1}^{N}{\left(y_{i}-\bar{y}\right)}^{2}}\sqrt{\sum_{i=1}^{N}{\left({\hat{y}}_{i}-\bar{\hat{y}}\right)}^{2}}}], \end{array}$$ (25)

where $\bar{y}$ and $\bar{\hat{y}}$ are the mean values of the real and predicted sequences, respectively. The larger the *R*^2^ is, the more variability is explained by the model.

Coefficient of efficiency:

$$\begin{array}{c} E_{sn}=1-\frac{\sum_{i=1}^{N}{\left(y_{i}-{\hat{y}}_{i}\right)}^{2}}{\sum_{i=1}^{N}{\left(y_{i}-\bar{y}\right)}^{2}} \end{array}$$ (26)

The larger the coefficient of efficiency is, the closer the predicted value is to the real value.

Mean absolute error:

$$\begin{array}{c} \text{MAE}=\frac{1}{N}\sum_{i=1}^{N}\left|y_{i}-{\hat{y}}_{i}\right|, \end{array}$$ (27)

**Fig 4:**
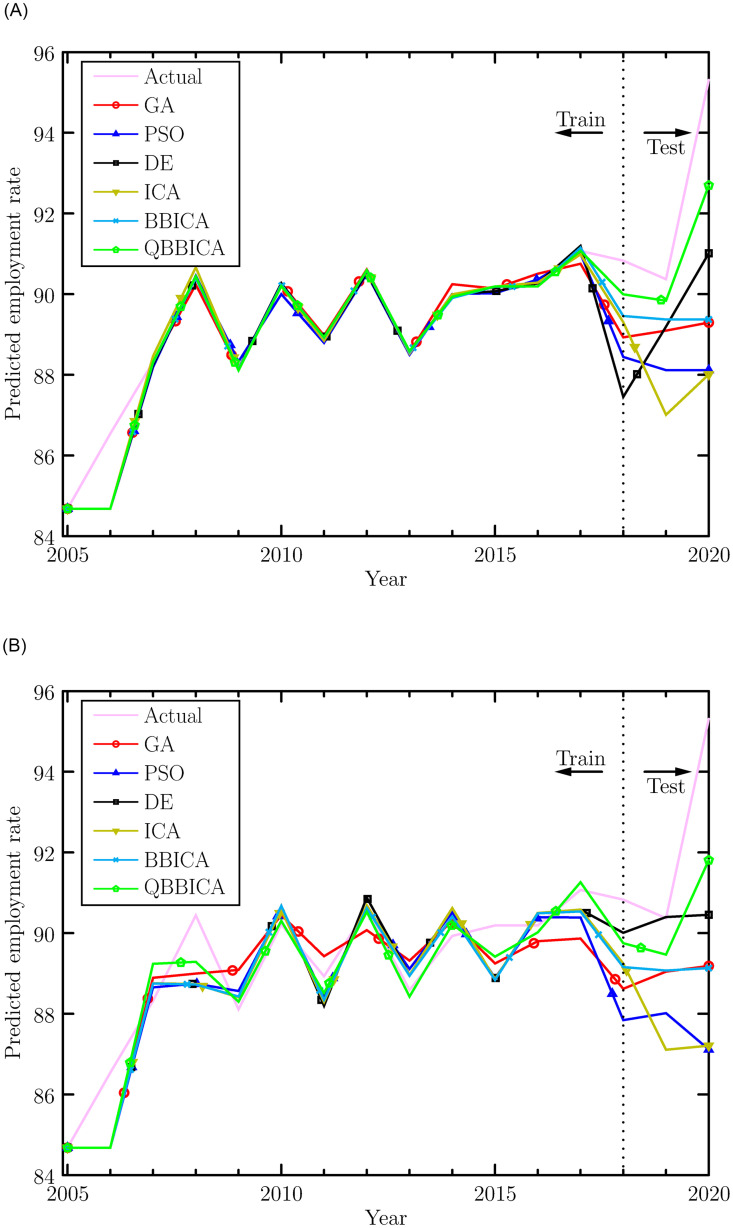
Prediction results of ReLu-FCM and Sig-FCM. (a) ReLu-FCM, (b) Sig-FCM.

**Fig 5:**
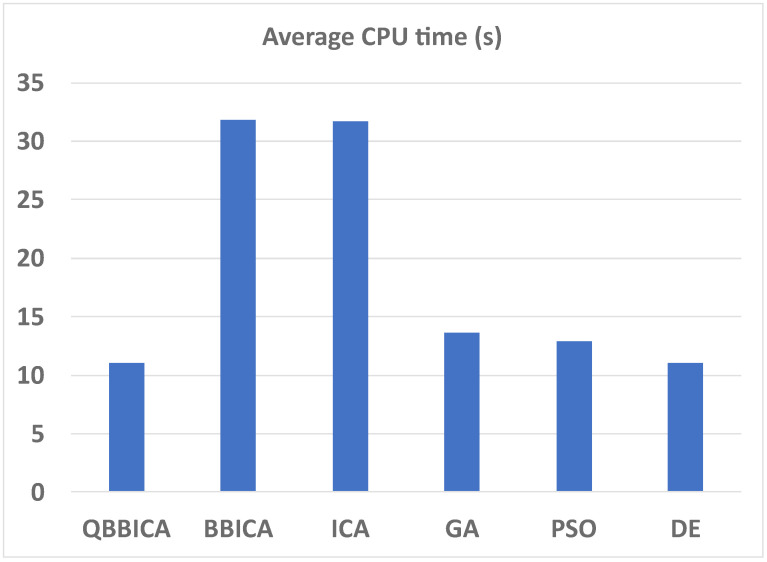
Average CPU time of QBBICA, BBICA, ICA, PSO, GA and DE in FCM training process.

**Fig 6:**
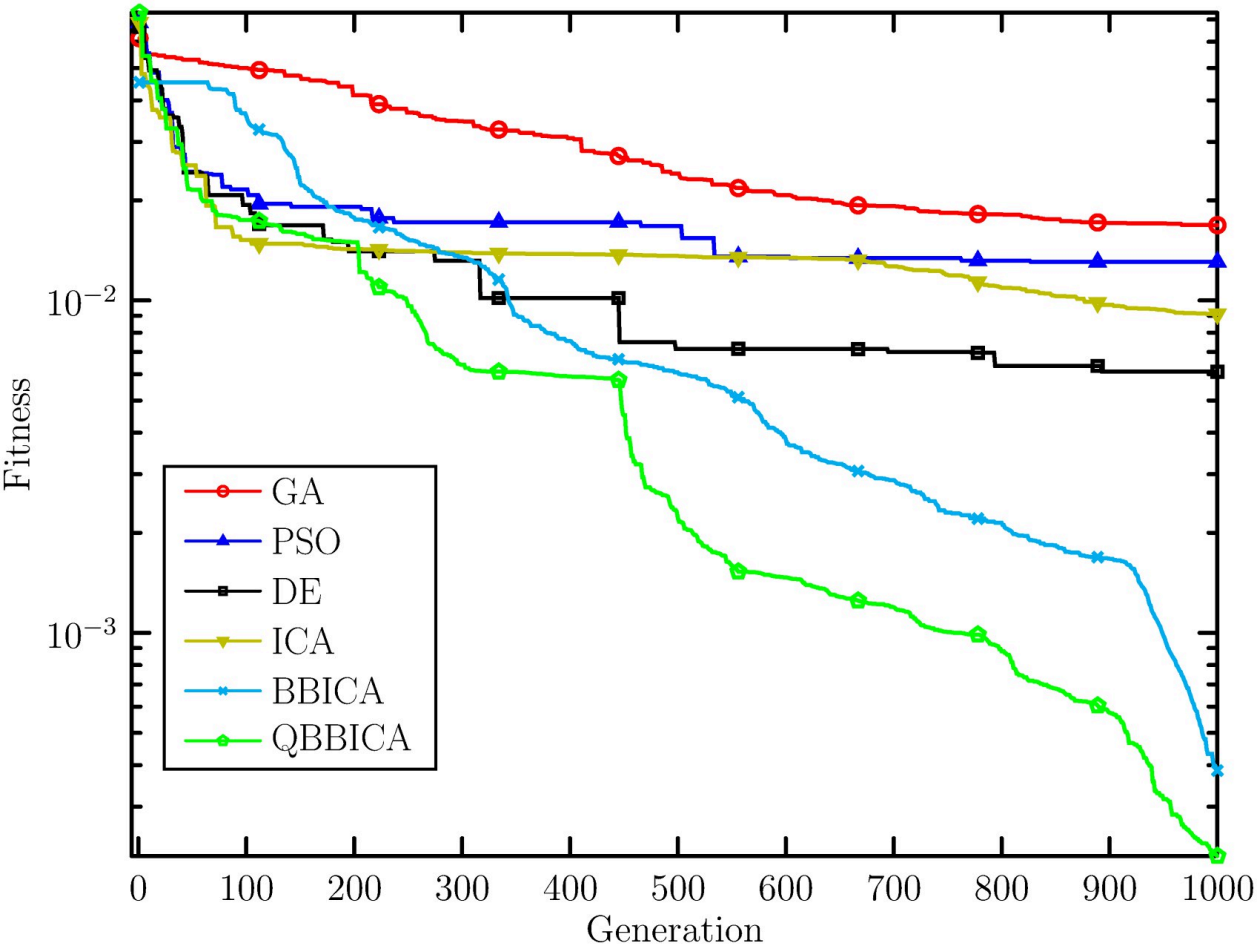
Fitness evolution curve of QBBICA, BBICA, ICA, PSO, GA and DE.

In the training phase, all the algorithms are run independently for **30** times. The employment rate data from 2005 to 2017 is used as the training set and that from 2018 to 2020 as the test set. The individual corresponding to the **best** fitness value in the 30 runs is taken as the final weight of the FCM. With the obtained weight, numerical prediction values are calculated using Eq (23). The prediction results from the training and test phases for ReLU-FCM and Sig-FCM are listed in Tables 6 and 7, and also, they are shown in Fig 4 for the sake of intuition. The performance indices are listed in Tables 8 and 9, respectively. It can be seen that the ReLU-FCM trained by QBBICA delivers better results than other FCMs in both prediction values and performance indices. The main reason for this is that QBBICA has a stronger search ability, which allows it to better balance exploration and exploitation. Therefore, QBBICA can find more appropriate weights of FCMs. Furthermore, the ReLU activation function has no saturation phenomenon, which enables ReLu-FCM to deliver better performance than Sig-FCM. On the other hand, to illustrate the computational efficiency of QBBICA and other referenced algorithms in FCM training process, the average cpu time in 30 runs is shown in Fig 5. Obviously, one can see that the average cpu time of expanded expended by QBBICA is less than ICA, BBICA, PSO and GA, but is competitive to DE. For a more straightforward comparison, the evolution curves of those algorithms are plotted in Fig 6. From Fig 6, it can be seen that QBBICA not only has a faster convergence speed but also a stronger search ability.

**Table 6:** Prediction results of ReLU-FCM trained by QBBICA, BBICA, ICA, RCGA, PSO and DE.

	Year	Actual	QBBICA	BBICA	ICA	PSO	GA	DE
Train	2005	84.68	**84.68**	84.68	84.68	84.68	84.68	84.6800
	2006	86.54	**84.68**	84.68	84.68	84.68	84.68	84.6800
	2007	88.32	**88.3180**	88.3002	88.4615	88.1898	88.3193	88.2419
	2008	90.44	**90.4419**	90.4015	90.6640	90.4548	90.2367	90.4090
	2009	88.11	**88.1115**	88.1306	88.1458	88.3276	88.1520	88.1529
	2010	90.21	**90.2005**	90.2243	90.1962	89.9994	90.2616	90.2727
	2011	88.93	**88.9303**	88.9203	88.8462	88.7924	88.9740	88.8517
	2012	90.59	**90.5905**	90.5807	90.5988	90.5021	90.6107	90.5244
	2013	88.59	**88.5881**	88.5609	88.5287	88.4979	88.5471	88.5306
	2014	89.93	**89.9346**	89.9045	89.9950	90.0028	90.2458	89.9977
	2015	90.19	**90.1902**	90.1865	90.1888	90.0216	90.1386	90.0717
	2016	90.19	**90.1926**	90.1894	90.2852	90.3621	90.5059	90.2670
	2017	91.07	**91.0720**	91.1503	90.9857	91.0014	90.7530	91.1956
Test	2018	90.83	**89.9950**	89.4580	89.3053	88.4427	88.9284	87.4515
	2019	90.37	**89.8360**	89.3722	87.0088	88.1153	89.0952	89.1950
	2020	95.31	**92.6969**	89.3722	88.0088	88.1153	89.2952	91.0132

**Table 7:** Prediction results of Sig-FCM trained by QBBICA, BBICA, ICA, RCGA, PSO and DE.

	Year	Actual	QBBICA	BBICA	ICA	PSO	GA	DE
Train	2005	84.68	84.68	84.68	84.68	84.68	84.68	84.6800
	2006	86.54	84.68	84.68	84.68	84.68	84.68	84.6800
	2007	88.32	89.2392	88.7506	88.7566	88.6554	88.8924	88.7566
	2008	90.44	89.2882	88.7260	88.7380	88.7466	88.9968	88.7380
	2009	88.11	88.2991	88.4276	88.4076	88.5643	89.0877	88.4076
	2010	90.21	90.3039	90.6749	90.5749	90.5912	90.4488	90.6749
	2011	88.93	88.5155	88.3467	88.2967	88.4324	89.4263	88.1967
	2012	90.59	90.5271	90.6203	90.6905	90.6524	90.0731	90.8805
	2013	88.59	88.4262	88.9403	88.9632	89.1113	89.3173	88.9632
	2014	89.93	90.2199	90.4105	90.6213	90.5154	90.3009	90.6213
	2015	90.19	89.4158	88.8382	88.8588	88.8818	89.2465	88.8588
	2016	90.19	90.0177	90.4904	90.4995	90.3952	89.7990	90.4995
	2017	91.07	91.2610	90.5363	90.5827	90.3869	89.8654	90.5827
Test	2018	90.83	89.7500	89.1580	89.2451	87.8427	88.6203	90.0078
	2019	90.37	89.4668	89.0722	87.1088	88.0153	89.0450	90.3982
	2020	95.31	91.8098	89.1325	87.2088	87.1153	89.1862	90.4539

**Table 8:** Performance indices of ReLU-FCM.

	QBBICA	BBICA	ICA	PSO	GA	DE
	Training					
RMSE	0.0035	0.0311	0.0967	0.1388	0.1790	0.049
*E*_*sn*_	1.0000	0.9990	0.9900	0.9793	0.9657	0.9121
*R* ^2^	1.0000	0.9991	0.9910	0.9809	0.9659	0.9505
MAE	0.0025	0.0228	0.0741	0.1248	0.1278	0.0193
	Test					
RMSE	1.6136	3.5654	4.7233	4.5661	3.7157	3.2279
*E*_*sn*_	0.4756	-1.5603	-3.4934	-3.1991	-1.7807	-1.0985
*R* ^2^	0.9988	0.1808	0.0001	0.1808	0.7216	0.685
MAE	1.3274	2.7692	4.0624	3.9456	3.0637	2.9501

**Table 9:** Performance indices of Sig-FCM.

	QBBICA	BBICA	ICA	PSO	GA	DE
	Training					
RMSE	0.5367	0.7590	0.7653	0.7638	0.8033	0.8856
*E*_*sn*_	0.6914	0.3827	0.3724	0.3748	0.3086	0.8029
*R* ^2^	0.7117	0.4909	0.4900	0.4628	0.3208	0.8192
MAE	0.4021	0.5961	0.6116	0.6116	0.7166	0.6906
	Test					
RMSE	2.1782	3.7701	5.1243	5.2161	3.8358	2.8436
*E*_*sn*_	0.0444	-1.8628	-4.2887	-4.4797	-1.9633	-0.6286
*R* ^2^	0.9993	0.0956	0.1500	0.9906	0.3966	0.2768
MAE	1.8278	3.0491	4.3158	4.5122	3.2195	1.9022

In order to demonstrate the advantage of FMC in prediction, the prediction results obtained by our proposed QBBICA-FCM is compared to those by BP neural network, linear regression (LR) model and grey GM(1,1) model, which are listed in Tables 10 and 11. From Table 10, one can see that the prediction value obtained by our QBBICA-FCM is more accurate than those by BP neural network, LR model and GM(1,1) model. Furthermore, the four performance indices of QBBICA-FCM are also better than those of BP, LR and GM(1,1). This proves that our proposed QBBICA-FCM can achieve more accurate prediction results. From the above experimental results and comparisons, one can draw a conclusion that our proposed method can give more accurate prediction results than the other methods.

**Table 10:** Prediction results comparison of QBBICA-FCM between BP, LR and GM(1,1).

	Year	Actual	QBBICA-FCM	BP	LR	GM(1,1)
Train	2005	84.68	**84.68**	**84.68**	**84.68**	**84.68**
	2006	86.54	**84.68**	**84.68**	**84.68**	88.07329
	2007	88.32	**88.3180**	88.5626	88.8343	88.3169
	2008	90.44	**90.4419**	88.6727	89.0873	88.5613
	2009	88.11	**88.1115**	88.3511	89.2250	88.8063
	2010	90.21	**90.2005**	88.4289	90.6067	89.0519
	2011	88.93	**88.9303**	88.7415	89.2037	89.2983
	2012	90.59	**90.5905**	90.6362	90.3146	89.5453
	2013	88.59	**88.5881**	88.5222	89.4168	89.7931
	2014	89.93	**89.9346**	89.9140	90.5428	90.0415
	2015	90.19	**90.1902**	90.1587	89.4545	90.2906
	2016	90.19	**90.1926**	90.2730	89.8932	90.5403
	2017	91.07	**91.0720**	90.3632	89.9910	90.7908
Test	2018	90.83	**89.9950**	89.5669	89.7703	91.0420
	2019	90.37	**89.8360**	90.3269	90.1614	91.2938
	2020	95.31	**92.6969**	90.3726	90.1866	91.5464

**Table 11:** Performance comparison of QBBICA-FCM and BP, LR and GM(1,1).

	QBBICA-FCM	BP	LR	GM(1,1)
	Train			
RMSE	0.5367	0.7956	0.7686	0.7032
*E*_*sn*_	0.6914	0.1790	-0.7247	0.6408
*R* ^2^	0.7117	0.5003	0.3670	0.7358
MAE	0.4021	0.4701	0.6799	0.6713
	Test			
RMSE	2.1782	2.9425	3.0230	2.2408
*E*_*sn*_	0.0444	-2.4051	-2.5973	-1.4016
*R* ^2^	0.9993	0.2212	0.2240	0.6745
MAE	1.8278	2.0812	2.1306	1.6331

## 6 Conclusion

In this paper, an improved FCM is proposed to predict the employment rate of university graduates. Two improvements are made in order to get more accurate prediction results. One is that the sigmoid activation function is replaced with the ReLu function. The other is that an improved ICA algorithm, called quasi-oppositional learning bare-bone ICA (QBBICA), is proposed to train the FCM. The QBBICA algorithm has a stronger search ability and can obtain more appropriate weights of the FCM. Experiments are conducted to predict the employment rate of graduates from Liren College, Yanshan University. Results show that our proposed method can give accurate prediction results.
